# Supplementary material for: Clinical Outcomes of HER2-Negative Metastatic Breast Cancer Patients in Italy in the Last Decade: Results of the GIM 13-AMBRA Study
Source: Cancers (Basel). 2023 Dec 25;16(1):117. doi: 10.3390/cancers16010117 (PMC10777910; doi:10.3390/cancers16010117)

Table S1 - Patients' and tumor's characteristics at diagnosis

	Luminal A	Luminal B	TNBC	Whole population
n (% of total)	386(41.10%)	408 (45.45%)	145 (15.44%)	939
Median age (range)	52.8 (20.8-82.1)	50.1 (17.6-82.2)	52.8 (27.4-85.3)	51.9 (50.6-52.9)
Mean Ki67% (95%CI)	10.48 (9.86-11.07)	35,8 (34.12-37.66)	53.84 (49.51-58.17)	24 (27.6-30.7)*
Node+ve (%)	245 (63.47%)	260(63.72%)	75 (51.72%)	580 (61.76%)
Grading 3 (%)	76 (19.6%)	195 (47.7%)	103 (71.03%)	474 (50.47%)

*n available =775

Table S2 – Outcome parameters according to subtypes (MANOVA)

Outcome parameter	Luminal A	Luminal B	TNBC	P value (α=0.05)
Median DFS (95% CI)	87.1 (75.3-91.7)	50.7 (46.4-56.5)	24.3 (21.6-29.2)	F-Ratio 33.73 P=0.00000
Median PFS 1st line (95% CI)	14.9 (12.7-17.0)	11.9 (11.0-13.2)	8.8 (6.7-10.2)	F-Ratio 1.73 P=0.178
Median PFS 2nd line (95% CI)	9.4 (8.1-10.7)	7.7 (6.8-8.2)	5.4 (4.3-6.5)	F-Ratio 4.30 P=0.014
Median TTC1 (95% CI)	13.2 (11.7-15.1)	11.8 (10.3-12.8)	7.7 (5.7-9.2)	F-Ratio 5.38 P=0.004
Median TTC2 (95% CI)	9.1 (7.7-10.5)	7.2 (6.3-8.1)	4.9 (3.7-6.2)	F-Ratio 4.72 P=0.009
Median OS * (95% CI)	9.9 (9.2-10.7)	6.9 (6.5-7.8)	3.6 (3.1-4.4)	F-Ratio 27.56 P=0.00000
Median OS from PD1** (95% CI)	35.2 (30.8-37.4)	28.9 (26.2-31.2)	18.5 16.0-20.1)	

*Median OS from primary tumor is expressed in years
**PD1=first progression

Figure S1 – Stage by subtype

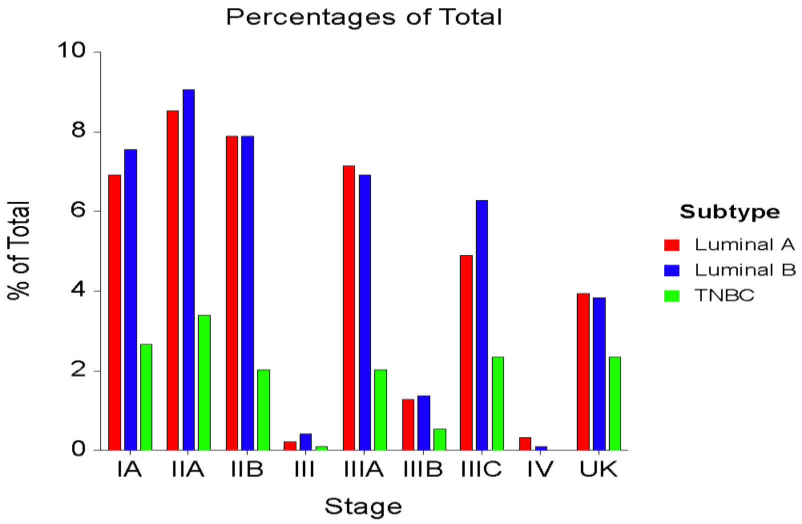

Supplement: Supplementary file 1 [file cancers-16-00117-s001.zip › cancers-2688671-supplementary.pdf]
